# Suspected Counterfeit M-30 Oxycodone Pill Exposures and Acute Withdrawals Reported from a Single Hospital — Toxicology Investigators Consortium Core Registry, U.S. Census Bureau Western Region, 2017–2022

**DOI:** 10.15585/mmwr.mm7329a2

**Published:** 2024-07-25

**Authors:** Emily Glidden, R. Matthew Gladden, Chris Dion, Meghan B. Spyres, Puja Seth, Kim Aldy, Desiree Mustaquim, Toxicology Investigators Consortium (ToxIC)

**Affiliations:** ^1^National Center for Injury Prevention and Control, CDC; ^2^Department of Medical Toxicology, Banner-University Medical Center Phoenix, Phoenix, Arizona; ^3^The University of Arizona College of Medicine-Phoenix, Phoenix, Arizona; ^4^American College of Medical Toxicology, Phoenix, Arizona.

**Summary**

**What is already known about this topic?**

Counterfeit prescription pill (counterfeit pill) availability has sharply increased in the United States and has increasingly been linked to overdose deaths.

**What is added by this report?**

Patients aged 15–34 years accounted for approximately two thirds of 143 suspected exposures to counterfeit pills containing fentanyl evaluated at a U.S. hospital. The majority of patients with exposures were hospitalized, 69% of whom were admitted to an intensive care unit. Substances in addition to fentanyl were detected in approximately 90% of exposures.

**What are the implications for public health practice?**

Outreach focusing on younger persons misusing prescription pills, improving access to harm reduction, and linking patients treated for overdoses in hospitals to substance use treatment might help prevent overdoses involving counterfeit pills.

## Abstract

Availability of counterfeit prescription pills (counterfeit pills) containing illegally made fentanyl, including counterfeit M-30 oxycodone (counterfeit M-30) pills, has risen sharply in the United States and has been increasingly linked to overdose deaths. In 2023, approximately 115 million counterfeit pills were seized in U.S. High Intensity Drug Trafficking Areas. However, clinical data on counterfeit pill–related overdoses are limited. Medical toxicology consultations during 2017–2022 from one U.S. Census Bureau Western Region hospital participating in the Toxicology Investigators Consortium Core Registry were analyzed. A total of 352 cases suspected to involve counterfeit M-30 pills, including 143 (40.6%) cases of fentanyl exposure and 209 (59.4%) cases of acute withdrawal were identified; consultations increased from three in 2017, to 209 in 2022. Patients aged 15–34 years accounted for 95 (67.4%) exposure cases. Among all patients with exposures, 81.1% were hospitalized, 69.0% of whom were admitted to an intensive care unit. Additional substances were detected in 131 (91.6%) exposures. Providing outreach to younger persons misusing prescription pills, improving access to and distribution of harm reduction tools including fentanyl test strips and naloxone, and promoting linkage of persons treated for overdose in hospitals to harm reduction and substance use treatment services are strategies to reduce morbidity associated with use of counterfeit M-30.

## Introduction

Broad distribution of counterfeit prescription pills (counterfeit pills) began in the United States in 2014.* Counterfeit pills are manufactured to mimic legitimate prescription drugs, but instead contain other drugs such as illegally made fentanyl (IMF). Counterfeit M-30 oxycodone (counterfeit M-30) accounts for the majority of counterfeit pills.^†^ Approximately 115 million counterfeit pills were seized by law enforcement agents in U.S. High Intensity Drug Trafficking Areas^§^ in 2023, representing approximately one half of all fentanyl seizures.^¶^ In 2022, an estimated six in 10 seized counterfeit pills contained a potentially lethal dose of fentanyl (≥2 mg).** A recent study found that 55.8% of overdose deaths with evidence of counterfeit pill involvement occurred in western jurisdictions; counterfeit M-30 was implicated in the majority of deaths involving oxycodone pills (*1*). However, despite well-documented proliferation and evidence of involvement of counterfeit pill–related incidents in overdose deaths, clinical data are limited. This study analyzed data reported to the Toxicology Investigators Consortium (ToxIC) Core Registry from a single hospital (hospital A), a facility operating in the U.S. Census Bureau Western Region, during 2017–2022 (*2*).

## Methods

### Data Collection

Medical toxicologists participating in the ToxIC Core Registry collect patient data from bedside consultations (e.g., patient or proxy interviews, physical examination, and ancillary data). Variables collected include patient demographic characteristics, exposures (i.e., specific drugs taken), clinical presentation (e.g., respiratory depression), treatments administered (e.g., naloxone), and outcomes (e.g., hospitalization) (*3*).

### Inclusion and Exclusion Criteria

Cases in the ToxIC Core Registry were identified as those in which the medical record mentioned 1) use of suspected counterfeit M-30, 2) symptomatic exposure to fentanyl (i.e., acute opioid overdose) or acute withdrawal from fentanyl, and 3) an administration route not typical for prescription fentanyl (i.e., nondermal). Of 986 hospital A cases initially identified, 505 (51.2%) were excluded because 1) laboratory testing data were not available to confirm fentanyl exposure, 2) the case was related to accidental or unintentional ingestion, or 3) the hospital visit or the clinical presentation was not directly related to the exposure (e.g., the patient was seen for an addiction medicine consultation). The remaining 481 (48.8%) cases were reviewed by medical toxicologists from hospital A. Additional cases were excluded if 1) the visit was determined to be unrelated to suspected counterfeit M-30 pills (43), 2) fentanyl was not detected either through urine drug screen (UDS) or gas chromatography–mass spectrometry (GC-MS) laboratory testing (51), or 3) oxycodone was detected through UDS or GC-MS laboratory testing (35). Cases in which oxycodone was detected with fentanyl were excluded because the patient might have purposefully used prescription M-30 pills (obtained either with or without a personal prescription) and fentanyl. This exclusion process left 352 (36%) cases within the analytical sample.

### Data Analysis

Data were analyzed descriptively, including patient demographic characteristics, route of suspected counterfeit M-30 administration, clinical presentation, clinical diagnosis, year of medical toxicology consultation, and treatment provided. Analyses were conducted using SAS (version 9.4; SAS Institute). This activity was reviewed by CDC, deemed not research, and was conducted consistent with applicable federal law and CDC policy.^††^

## Results

### Exposure and Acute Withdrawal Consultations

During 2017–2022, a total of 352 suspected counterfeit M-30 pill–related cases were identified, including 143 exposures (40.6%) and 209 acute withdrawals (59.4%) (Table 1). Exposures increased from three in 2017 to 53 in 2022. Acute withdrawals first occurred in 2019 (seven) and increased to 38 in 2021, before sharply increasing to 156 in 2022.

**TABLE 1:** Epidemiologic characteristics of medical toxicology consultations involving suspected counterfeit M-30 oxycodone pill exposures and acute withdrawals reported by hospital A — Toxicology Investigators Consortium Core Registry, U.S. Census Bureau Western Region, 2017–2022

Characteristic	No. (%)		
	All cases	Exposures	Withdrawals
**Total**	**352 (100.0)**	**143 (40.6)**	**209 (59.4)**
**Patient gender identity**			
Female	**161 (45.7)**	41 (28.7)	120 (57.4)
Male	**191 (54.3)**	102 (71.3)	89 (42.6)
Patient age group, yrs*			
12–14	**3 (0.9)**	2 (1.4)	1 (0.5)
15–17	**40 (11.4)**	32 (22.7)	8 (3.8)
18–24	**54 (15.4)**	25 (17.7)	29 (13.9)
25–34	**127 (36.3)**	38 (27.0)	89 (42.6)
35–44	**65 (18.6)**	28 (19.9)	37 (17.7)
≥45	**61 (17.4)**	16 (11.3)	45 (21.5)
Missing	**2**	2	0
**Patient race and ethnicity^†,§^**			
Black or African American	**38 (10.8)**	17 (11.9)	21 (10.0)
White	**148 (42.0)**	43 (30.1)	105 (50.2)
Hispanic or Latino	**126 (35.8)**	57 (39.9)	69 (33.0)
Other or multiple races	**15 (4.3)**	7 (4.9)	8 (3.8)
Unknown	**25 (7.1)**	19 (13.3)	6 (2.9)
**Route of administration^¶^**			
Ingestion	**59 (16.9)**	44 (31.2)	15 (7.2)
Inhalation or smoking	**168 (48.0)**	36 (25.5)	132 (63.2)
Intranasal or snorting	**20 (5.7)**	14 (9.9)	6 (2.9)
Other	**3 (0.9)**	2 (1.4)	1 (0.5)
Unknown or not reported	**100 (28.6)**	45 (31.9)	55 (26.3)
Missing	**2**	2	0
**Year**			
2017	**3 (0.9)**	3(2.1)	0 (—)
2018**	**10 (2.8)**	10 (7.0)	0 (—)
2019	**22 (6.3)**	15 (10.5)	7 (3.3)
2020	**28 (8.0)**	20 (14.0)	8 (3.8)
2021	**80 (22.7)**	42 (29.4)	38 (18.2)
2022	**209 (59.4)**	53 (37.1)	156 (74.6)

**Abbreviation:** GC-MS = gas chromatography–mass spectrometry.

* Age group percentages were calculated by dropping missing values (two) from the denominator.

**^†^** Distribution by race and ethnicity was comparable to that of the residential population of the county where the hospital provides services, based on county-level U.S. Census Bureau data.

^§^ Persons of Hispanic or Latino (Hispanic) origin might be of any race but are categorized as Hispanic; all racial groups are non-Hispanic. Patients who identified as other or multiple races included American Indian or Alaskan Native, Asian, Australian Aboriginal, Native Hawaiian or Pacific Islander, or more than one race.

^¶^ Route of administration percentages were calculated by dropping missing values (two) from the denominator.

** In July 2018, hospital A implemented changes in toxicological testing for fentanyl that might have resulted in improved detection of cases. The previous methodology for GC-MS detection of fentanyl was replaced with a system that has a lower limit of detection; urine drug screening for fentanyl was instituted in addition to GC-MS.

### Patient Demographic Characteristics and Routes of Administration

Patients with exposures (143) were predominantly male (71.3%). Of the 141 exposures with age data, patients aged 15–17 (32), 18–24 (25), and 25–34 (38) years accounted for approximately two thirds (67.4%) of exposures.

Among exposures, the most reported routes of administration were ingestion (44; 31.2%) and inhalation (36; 25.5%). Among acute withdrawals, inhalation was the most common route (132; 63.2%). Where data were available (243), route of administration also varied by age group, with 37.1% of patients aged 15–17 years reporting ingestion (versus 24.3% overall) and 79.8% of patients aged 25–34 years reporting inhalation (versus 67.9% overall) (Figure).

**FIGURE:**
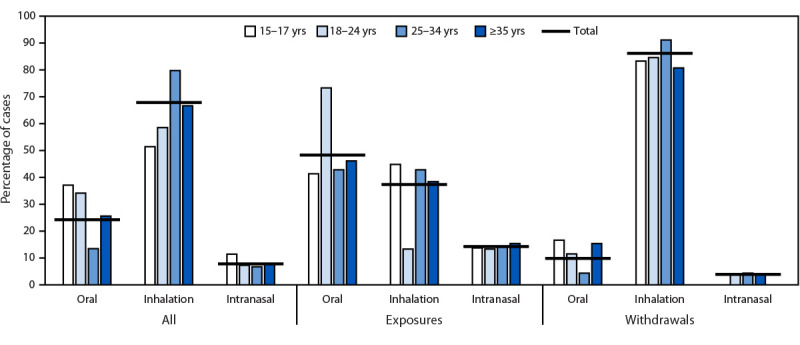
Medical toxicology consultations involving suspected counterfeit M-30 oxycodone pill exposures and acute withdrawals reported by hospital A (N = 243), by age group and route of administration — Toxicology Investigators Consortium Core Registry, U.S. Census Bureau Western Region, 2017–2022* * Cases presented (all cases = 243; exposures = 91; and withdrawals = 152) do not include patients aged 12–14 years (three) or cases with missing age data (two). Consultations in which route of administration data was not ingestion, not inhalation, not intranasal, not reported, unknown, or missing (all cases = 105; exposures = 49; and withdrawals = 56) are not reported and have been removed from the denominator; therefore, percentages only represent cases with route of administration data present in the medical record. One case was missing both age and route of administration data. Age groups for this figure are 15–17 years (35), 18–24 years (41), 25–34 years (89), and ≥35 years (78).

### Clinical Signs and Outcomes

Of the 143 patients with exposures, the majority were hospitalized (116; 81.1%); 80 (69.0%) of these patients were admitted to an intensive care unit (Table 2). Among patients with exposures, 74.1% had clinical signs of an opioid toxidrome; 56.6% of respiratory depression or bradypnea, and 38.5% of coma or central nervous system depression. Overall, two deaths were reported during hospitalization, both in patients with exposures (1.4%).

**TABLE 2:** Clinical and laboratory characteristics of medical toxicology consultations involving suspected counterfeit M-30 oxycodone pill exposures and acute withdrawals reported by hospital A — Toxicology Investigators Consortium Core Registry, U.S. Census Bureau Western Region, 2017–2022

Characteristic*	No. (%)		
	All cases	Exposures	Withdrawals
**Total**	**352 (100.0)**	**143 (40.6)**	**209 (59.4)**
**Highest level of care received**			
Hospital admission^†^	**285 (81.0)**	116 (81.1)	169 (80.9)
ICU	**106 (37.2)**	80 (69.0)	26 (15.4)
Non-ICU	**179 (62.8)**	36 (31.0)	143 (84.6)
Emergency department	**9 (2.6)**	4 (2.8)	5 (2.4)
Unknown	**58 (16.5)**	23 (16.1)	35 (16.7)
**Discharged alive**			
Yes	**350 (99.4)**	141 (98.6)	209 (100.0)
No	**2 (0.6)**	2 (1.4)	0 (—)
**Clinical signs**			
Opioid toxidrome	**273 (77.6)**	106 (74.1)	167 (79.9)
Respiratory depression/Bradypnea	**97 (27.6)**	81 (56.6)	16 (7.7)
Coma/Central nervous system depression	**55 (15.6)**	55 (38.5)	0 (—)
**Laboratory findings^§^**			
Fentanyl with any additional substance of interest^¶^	**322 (91.5)**	131 (91.6)	191 (91.4)
Amphetamine/Methamphetamine**	**233 (66.2)**	78 (54.5)	155 (74.2)
Benzodiazepine^††^	**60 (17.0)**	34 (23.8)	26 (12.4)
Cocaine^§§^	**18 (5.1)**	15 (10.5)	3 (1.4)
Buprenorphine^¶¶^	**9 (2.6)**	3 (2.1)	6 (2.9)
Methadone***	**26 (7.4)**	4 (2.8)	22 (10.5)
Other opioids^†††^	**22 (6.3)**	4 (2.8)	18 (8.6)
Fentanyl with no other opioids, stimulants, or benzodiazepines	**30 (8.5)**	12 (8.4)	18 (8.6)
**Naloxone administration^§§§^**			
Any naloxone administration	**142 (40.3)**	115 (80.4)	27 (12.9)
Naloxone drip infusion	**22 (15.5)**	22 (19.1)	0 (—)
Intravenous	**27 (19.0)**	20 (17.4)	7 (25.9)
Intranasal	**20 (14.1)**	16 (13.9)	4 (14.8)
Intramuscular	**22 (15.5)**	18 (15.7)	4 (14.8)
Unknown route	**51 (35.9)**	39 (33.9)	12 (44.4)
No naloxone administration	**210 (59.7)**	28 (19.6)	182 (87.1)
**Nonpharmacological treatment administered**			
Intubation/Ventilatory management	**60 (17.0)**	51 (35.7)	9 (4.3)
Cardiopulmonary resuscitation	**7 (2.0)**	6 (4.2)	1 (0.5)

**Abbreviations:** GC-MS = gas chromatography–mass spectrometry; ICU = intensive care unit; UDS = urine drug screen.

* More than one clinical sign, laboratory finding, or treatment administered could be reported per case.

^†^ Among hospitalized patients, non-ICU hospital admissions were to a general inpatient unit (178; 62.5%) or an inpatient psychiatric facility (19; 6.7%). ICU and non-ICU percentages are calculated using corresponding hospital admissions numbers as denominators.

^§^ All percentages are calculated using the respective group numbers (all cases = 352; exposures = 143; and withdrawals = 209) as denominators.

^¶^ Fentanyl detected in UDS analysis (291; 82.7%) and GC-MS analysis (174; 49.4%).

** Amphetamine/methamphetamine detected in UDS analysis (208; 59.1%) and GC-MS analysis (159; 45.2%).

^††^ Benzodiazepine detected in UDS analysis (55; 15.6%) and GC-MS analysis (23; 6.5%).

^§§^ Cocaine or cocaine metabolites detected in UDS analysis (16; 4.5%) and GC-MS analysis (10; 2.8%).

^¶¶^ Buprenorphine detected in UDS analysis (nine; 2.6%) and GC-MS analysis (one; 0.28%).

*** Methadone detected in UDS analysis (16; 4.5%) and GC-MS analysis (18; 5.1%). Methadone is prescribed for both chronic pain relief and as a medication for opioid use disorder. The intended use of methadone could not be determined using available data.

^†††^ Opioids (other than fentanyl, buprenorphine, or methadone) detected in UDS analysis (22; 6.3%) and GC-MS analysis (10; 2.8%).

^§§§^ Naloxone route of administration (i.e., drip infusion, intravenous, intranasal, intramuscular, and unknown route) percentages are calculated among cases with any naloxone administration. Naloxone administration was identified in the case narrative or the respective treatment field.

### Detection of Substances

At least one substance other than fentanyl^§§^ was detected among the majority of patients (322; 91.5%). The substances most commonly detected with fentanyl were amphetamine/methamphetamine (66.2%), benzodiazepines (17.0%), and cocaine (5.1%).

### Naloxone Administration

Among patients with exposures who received naloxone (80.4%), a naloxone drip infusion was administered in 19.1% of patients. These patients included nine of 44 (20.5%) of those with ingestion exposures.

## Discussion

Consultations for exposure to and acute withdrawal from suspected counterfeit M-30 pills increased during 2017–2022 at hospital A, from three in 2017 to 209 in 2022. Approximately two thirds of exposures occurred among patients aged 15–34 years. The majority of patients with suspected counterfeit M-30 exposure who were admitted to a hospital were admitted to an intensive care unit. Ingestion and inhalation were common routes of administration, and additional substances, including amphetamine/methamphetamine, benzodiazepines, and cocaine were frequently detected with fentanyl. These findings suggest that additional efforts are needed to prevent and reduce harm from use of counterfeit pills, especially among youths and young adults.

These findings are consistent with a broader trend that has been observed nationally and regionally. Overdose deaths with evidence of counterfeit pill exposure increased from 2.0% to 4.7% during July 2019–December 2021 in the United States, largely driven by a tripling in western jurisdictions (*1*). Further, 57.1% of decedents were aged <35 years, and 39.5% reported inhalation as the route of administration (*1*).

In this report, approximately one in three (31.2%) suspected counterfeit M-30 pill exposures occurred through ingestion. Persons who ingest pills might believe they are using a legitimate prescription drug. Unsuspected exposure to IMF is concerning because of its high potency and the possibility of rapid overdose (*4*). IMF-related overdoses involving ingested pills might require naloxone drip infusion or extended observation because of delayed, recurrent toxicity, as fentanyl continues to be gradually absorbed (*5*). In this analysis, approximately one in five patients with ingestion exposure was administered a naloxone drip infusion.

Evidence that some persons purposefully use counterfeit pills with IMF exists. The majority of persons accessing syringe service programs in Washington reported knowing their pill contained IMF.^¶¶^ Some reports suggest that persons using drugs in the West might be shifting from injecting heroin to intentionally inhaling counterfeit pills with IMF because of cost, convenience, difficulties with injection, and reduced stigma (*6*). These findings are likely part of a larger nationwide shift toward inhaling or smoking IMF and away from injecting IMF (*7*).

In this study, detection of substances other than fentanyl was common. Co-exposure can mask opioid-related signs, complicating treatment. Further, sympathomimetic signs might appear after naloxone administration with stimulant co-exposures, and sedation could persist after naloxone administration with benzodiazepine co-exposures, both potentially requiring further medical intervention (*8*,*9*).

Approximately two thirds of exposures involved persons aged 15–34 years. Overdose deaths involving IMF among those aged 10–19 years sharply increased across 31 states during July 2019–December 2021, with evidence of counterfeit pills among one quarter of deaths (*10*). Easy access to counterfeit pills through sources such as social media*** might be increasing exposure to IMF and risk of overdose death among youths and young adults (*10*).

### Limitations

The findings in this report are subject to at least six limitations. First, descriptions of the drug products used are based on patient self-report and were not verifiable. Second, in July 2018, hospital A implemented changes in laboratory methodology to improve detection of fentanyl in patient specimens; this improvement in detection could account for some of the increase in cases identified during the study period. Third, less severe cases are unlikely to require a medical toxicology consultation, biasing results toward more severe or complex clinical presentations. Fourth, data within the ToxIC Core Registry on outcome, beyond clinical death, have improved over time but were limited during the study period. Fifth, hospital A outpatient addiction medicine services were discontinued in March 2022; after this date, patients experiencing acute withdrawal might have been more likely to be referred for a medical toxicology consultation, accounting for some of the increase in cases identified in this analysis. Finally, data were from a single site and are not generalizable.

### Implications for Public Health Practice

Linking persons treated in hospitals for an overdose to evidence-based substance use treatment,^†††^ and increasing outreach and linkage to care among youths and young adults who use diverted prescription pills (i.e., pills obtained without legitimate prescription) or who purposefully use IMF, could help prevent and minimize further harm associated with exposure to counterfeit pills. Other critical actions include improving access to harm reduction tools, such as fentanyl test strips to reduce unintentional exposure to IMF, and naloxone to reverse opioid overdose.^§§§^ CDC recently launched support for surveillance activities through the Overdose Data to Action Program to provide laboratory testing of biologic specimens from patients with signs and symptoms of overdose, as well as testing of drug products and paraphernalia, to detect and track substances involved in drug overdoses. These data can help communities identify, tailor, and scale-up drug overdose prevention programs and policies.^¶¶¶^ Increased awareness among clinicians, public health and public safety officials, and community-based organizations is needed to implement prevention strategies to reduce overdoses involving counterfeit pills.

## Footnotes

*
https://www.dea.gov/press-releases/2016/07/22/dea-report-counterfeit-pills-fueling-us-fentanyl-and-opioid-crisis

†
https://www.dea.gov/sites/default/files/2021-05/Counterfeit%20Pills%20fact%20SHEET-5-13-21-FINAL.pdf

§
https://www.whitehouse.gov/ondcp/grant-programs/hidta/

¶
https://nida.nih.gov/news-events/news-releases/2024/05/over-115-million-pills-containing-illicit-fentanyl-seized-by-law-enforcement-in-2023

**
https://www.dea.gov/alert/dea-laboratory-testing-reveals-6-out-10-fentanyl-laced-fake-prescription-pills-now-contain

†† 45 C.F.R. part 46; 21 C.F.R. part 56; 42 U.S.C. Sect. 241(d), 5 U.S.C. Sect. 552a, 44 U.S.C. Sect. 3501 et seq.

§§ Detection of a drug through UDS indicates that the person has recently used the drug and cannot be interpreted as meaning that the patient used the drug at the same time as fentanyl or that the drug contributed to the toxidrome or acute withdrawal symptoms treated by hospital physicians.

¶¶
https://adai.uw.edu/wordpress/wp-content/uploads/ssp-health-survey-2021.pdf

***
https://www.dea.gov/sites/default/files/2022-03/20220208-DEA_Social%20Media%20Drug%20Trafficking%20Threat%20Overview.pdf

†††
https://www.cdc.gov/overdose-prevention/hcp/clinical-guidance/linkage-to-care.html?CDC_AAref_Val%20=%20https://www.cdc.gov/drugoverdose/featured-topics/linkage-to-care.html

§§§
https://www.cdc.gov/overdose-prevention/php/od2a/harm-reduction.html

¶¶¶
https://www.cdc.gov/overdose-prevention/php/od2a/surveillance.html
